# Supplementary material for: Deletion upstream of MAB21L2 highlights the importance of evolutionarily conserved non-coding sequences for eye development
Source: Nat Commun. 2024 Oct 26;15:9245. doi: 10.1038/s41467-024-53553-2 (PMC11511899; doi:10.1038/s41467-024-53553-2)

Deletion upstream of *MAB21L2* highlights the importance of evolutionarily conserved non-coding sequences for eye development

Supplementary information

Contents

Supplementary Methods 3

Statistical analyses of zebrafish mutant lines 3

Supplementary Data 5

Family 2: Genetic findings from whole exome sequencing 5

Supplementary Tables 5

Supplementary Table 1: Relative locations of the Family 2 deletion and conserved elements (CEs) in human, mouse, chicken, *Xenopus tropicalis*, and zebrafish. 5

Supplementary Table 2: gRNA target sites in the *Xenopus tropicalis* genome 7

Supplementary Table 3: Primer sequences for genotyping the targeted regions in the *Xenopus tropicalis* 7

Supplementary Table 4: Overview of the primers used for *mab21l2* RNA probe generation from *Xenopus tropicalis* 7

Supplementary Figures 8

Supplementary Figure 1: Analysis of SNP array data and runs of homozygosity surrounding the 113.58kb chromosome 4 deletion in Family 2. 8

Supplementary Figure 2: Identification of 15 non-exonic conserved elements within the 113.58kb deletion of Individual III.5 (Family 2). 10

Supplementary Figure 3: Histograms of relative fold change in expression of *mab21l2* in wild-type and homozygous *mab21l2*^{mw715} fish. 12

Supplementary Figure 4: ChIP-seq data from murine stem cells showing OTX2 binding of Conserved Elements 13 and 14. 13

Supplementary Figure 5: Evidence of regulatory elements within the region of the deletion identified in Family 2. 15

Supplementary Figure 6: CRISPR/Cas9 genome editing strategy used to generate mosaic mutant *Xenopus tropicalis* tadpoles. 17

Supplementary Figure 7: Comparison of retinal layering in a *CE14* crispant injected and non-injected eyes. 19

Supplementary References 20

Supplementary Note 21

Supplementary Figure 6B (uncropped gel image). 21

Supplementary Methods

Statistical analyses of zebrafish mutant lines

For lens diameter and overall eye size measurements (Figures 2B-C), the embryos were photographed in the lateral (24- and 48hpf) or dorsal (72hpf) positions. Measurements were performed with ZEISS ZEN microscope software (Carl Zeiss) for 17 wild-type (WT) and 18 mutant eyes (at 24hpf), 22 WT and 30 mutant eyes (at 48hpf) and 29 WT and 30 mutant eyes (at 72hpf). A two-tailed t-test for two independent samples was used to determine whether there is a significant difference between the means of the lens diameter or eye width measurements in WT and *mab21l2*^{mw715} homozygous groups at indicated time points. The standard error of means (SEM) was calculated to determine the variance within each group. For the lens diameter measurements, the means were calculated as 285.7±8.1µm (24hpf), 416.3±9.7µm (48hpf) and 454.9±7.6µm (72hpf) for WT and 250.5±4.4µm (24hpf), 396.8±6.4µm (48hpf) and 446.5±8µm (72hpf) for homozygotes, assuming the 95% confidence level. Similar calculations were performed for eye width: means were 798.4±15.5µm (24hpf), 1119.5±32.8µm (48hpf) and 1224.5±16µm (72hpf) for WT and 806.9±22.7µm (24hpf), 1098.7±24.2µm (48hpf) and 1198.5±16µm (72hpf) for homozygotes. To determine the effect size of the difference between WT and homozygous groups, Cohen's d was computed for all WT and homozygous groups. For the lens diameter measurements, Cohen's d coefficient (d) was 2.8 (24hpf), 1.0 (48hpf) and 0.28 (72hpf), indicating large effect size for both early stages and moderate effect size for 72hpf. This result suggests that the difference in lens diameter at 24hpf and 48hpf is statistically significant. For eye width measurements, d was -0.2 (24hpf), 0.30 (48hpf) and 0.59 (72hpf). Small to moderate effect size indicates a relatively minor difference between means of the eye widths in each pair of measurements.

For qPCR data (Supplementary Figure 3), the total fold changes were estimated using the $2^{-\Delta\Delta Ct}$ method (with *beta-actin* expression as a normalizer) and the mean and standard error of means (SEM) were calculated for WT and homozygous samples. Technical replicates were averaged into a single value for each biological replicate prior to calculating the ΔCt. ΔCt was determined by subtracting average *actin* Cq value from *mab21l2* value; ΔCt values for WT samples at each time point were averaged to generate the value used in future calculations. ΔΔCt was calculated by subtracting average WT ΔCt for each time point from the ΔCt value for each biological replicate. Fold change was then calculated by applying $2^{-\Delta\Delta Ct}$ for each biological replicate. The two-tailed unpaired t-test was applied to calculate statistical significance. For homozygous embryos, the mean and SEM values were as follows: 0.7537 (SEM=0.0839), 0.8768 (SEM=0.1324), and 0.8219 (SEM=0.0710) for 24-, 48-, and 72hpf eye samples, correspondingly; 0.4041 (SEM=0.0307) and 0.2509 (SEM=0.0459) for 48- and 72hpf midbrain samples, correspondingly; and 0.6921 (SEM=0.0867), 0.7137 (SEM=0.0908), 0.9831 (SEM=0.1612), and 0.9492 (SEM=0.1915) for 20-, 24-, 48-, and 72hpf whole embryos, correspondingly. For WT embryos, the mean and SEM values were as follows: 1.0724 (SEM=0.1845), 1.0036 (SEM=0.0504), and 1.0041 (SEM=0.0524) for 24-, 48-, and 72hpf eye samples, correspondingly; 1.0034 (SEM=0.0466) and 1.0122 (SEM=0.1088) for 48- and 72hpf midbrain samples, correspondingly; and 1.0064 (SEM=0.0788), 1.0142 (SEM=0.1208), 1.0083 (SEM=0.0940), and 1.0588 (SEM=0.2497) for 20-, 24-, 48-, and 72hpf whole embryo samples, correspondingly.

Supplementary Data

Family 2: Genetic findings from whole exome sequencing

Whole exome sequencing of Individual III.5 (Family 2) identified a stop codon variant in *PIEZO2* (NM_022068, exon38, c.G5855A:p.[Trp1952*], chr18:10,704,456 [hg19], chr18:10,704,458 [hg38]), which was not maternally inherited (paternal DNA unavailable). The variant is absent from gnomAD v4.1.0. Previously, a single heterozygous missense variant in *PIEZO2* was tentatively implicated as a possible causal variant in a patient with isolated microphthalmia[1]. However, as loss-of-function (LoF) variants in this gene appear to be pathogenic only in the biallelic state (gene constraints metrics regarding LoF variants according to gnomAD v4.1.0: pLI=0, LOEUF=0.56) and are associated with motor and skeletal phenotypes[2], we considered this variant unlikely to explain the phenotype of Individual III.5. The scripts used to annotate and filter Family 2 WES data are provided in a separate folder (Supplementary Software 1).

Supplementary Tables

Supplementary Table 1: Relative locations of the Family 2 deletion and conserved elements (CEs) in human, mouse, chicken, *Xenopus tropicalis*, and zebrafish.

CEs 7, 8 and 9 contain the regulatory sequences Ma, Mb and Mc, respectively, identified by Tsang *et al.*[3] While CEs 4 and 5 are conserved in *Xenopus tropicalis*, they do not map to the vicinity of *mab21l2* and so are not listed.

Feature	Human (hg19)	Mouse (mm10)	Chicken (galGal6)	*Xenopus tropicalis* (xenTro9)	Zebrafish (GRCz11)
MAB21L2	4:151,503,077-151,505,845	3:86,545,581-86,548,283	4:33,007,990-33,009,429	1:47,346,416-47,349,141	1:24,076,286-24,078,098
CNV	4:151,370,118-151,483,697	3:86,565,054-86,628,286	4:32,960,380-32,988,495	1:47,366,984-47,405,406	1:24,032,155-24,064,147
CE1	4:151,378,979-151,379,314	3:86,626,038-86,626,364	4:32,960,773-32,961,088	N/A	N/A
CE2	4:151,381,225-151,381,449	3:86,624,881-86,625,097	N/A	N/A	N/A
CE3	4:151,407,162-151,407,292	N/A	N/A	N/A	N/A
CE4	4:151,410,152-151,410,335	N/A	N/A	N/A	N/A
CE5	4:151,410,386-151,410,727	N/A	N/A	N/A	N/A
CE6	4:151,412,674-151,413,028	3:86,605,108-86,605,431	4:32,968,790-32,969,132	N/A	N/A
CE7	4:151,416,673-151,417,093	3:86,602,352-86,602,784	4:32,970,351-32,970,681	1:47,389,194-47,389,376	1:24,053,541-24,053,736
CE8	4:151,418,673-151,419,605	3:86,599,912-86,600,870	4:32,972,597-32,973,557	1:47,386,569-47,387,336	N/A
CE9	4:151,446,445-151,446,701	3:86,589,474-86,589,723	N/A	N/A	N/A
CE10	4:151,453,193-151,453,960	3:86,583,275-86,584,044	4:32,979,936-32,980,720	1:47,383,743-47,384,461	1:24,059,514-24,059,930
CE11	4:151,455,895-151,456,111	3:86,581,851-86,581,990	4:32,982,597-32,982,796	1:47,381,773-47,381,940	N/A
CE12	4:151,464,211-151,464,504	3:86,575,429-86,575,718	4:32,985,265-32,985,481	N/A	N/A
CE13	4:151,467,297-151,468,022	3:86,572,365-86,573,077	4:32,986,640-32,987,419	1:47,378,366-47,378,871	N/A
CE14	4:151,468,356-151,469,080	3:86,571,403-86,572,099	4:32,987,762-32,988,495	1:47,376,224-47,376,764	N/A
CE15	4:151,483,297-151,483,432	3:86,565,291-86,565,426	N/A	N/A	N/A

Supplementary Table 2: gRNA target sites in the *Xenopus tropicalis* genome

gRNA name	Target Sequence	XenTro9 Coordinates	Efficiency
CE14	GCTCAATAATAGAGGGATTAAGG	chr1:47,376,296-47,376,318	45%
non-CE	CGCGCAAAGGATGGGTCGGGGGG	chr1:47,366,797-47,366,819	80%
altR del1	ATTTTTCAACACCTGTGATCAGG	chr1:47,366,984-47,367,003	60%
altR del2	AATTCTTTGAGACTAACTTTAGG	chr1:47,405,387-47,405,406	22%

Supplementary Table 3: Primer sequences for genotyping the targeted regions in the *Xenopus tropicalis*

Primer Set	Forward Primer	Reverse Primer
CE14	GAGATTGGCAGCAAAATGGT	GGGTTCTGAAAGGGACACAA
non-CE	TGTAAGGGGAGGATGACTGC	TTGCAAAGACGTGAATGCTC
altR del1	AAAGGCAGCAGAATCTTTCCT	GCCTAGCCTATGGGTAAGAAGC
altR del2	TGAGAGAAGGAGCAGAACGTC	TGCTGCTGTACGGAGAATCA

Supplementary Table 4: Overview of the primers used for *mab21l2* RNA probe generation from *Xenopus tropicalis*

Probe	Forward Primer coupled with T7 promoter	Reverse Primer coupled with T3 promoter
mab21l2	TAATACGACTCACTATAGGGTGAAGATGCGCCTATGAGTG	AATTAACCCTCACTAAAGGGGCTTGCCAGTCAGGGAGTAG

Supplementary Figures

Supplementary Figure 1: Analysis of SNP array data and runs of homozygosity surrounding the 113.58kb chromosome 4 deletion in Family 2.

A: SNP array B allele frequency and Log R ratio for Family 2 Individual III.5 (proband) and II.2 (mother). The region of the 113.58kb deletion is highlighted for III.5 (pink) and II.2 (orange) and indicated by black arrows. The deletion is located within a larger run of homozygosity in the proband (green highlight). **B:** Runs of homozygosity (ROHs) >500kb in the vicinity of the 113.58kb deletion. ROHs are indicated for 1000 Genomes Project participants (blue bars, 500 individuals assessed), III.5 (green bars), and II.2 (red bars). An ROH overlapping or adjacent to the 113.58kb deletion is indicated by a red box, being larger in the proband (III.5, green) than the mother (II.2, red).

Supplementary Figure 1

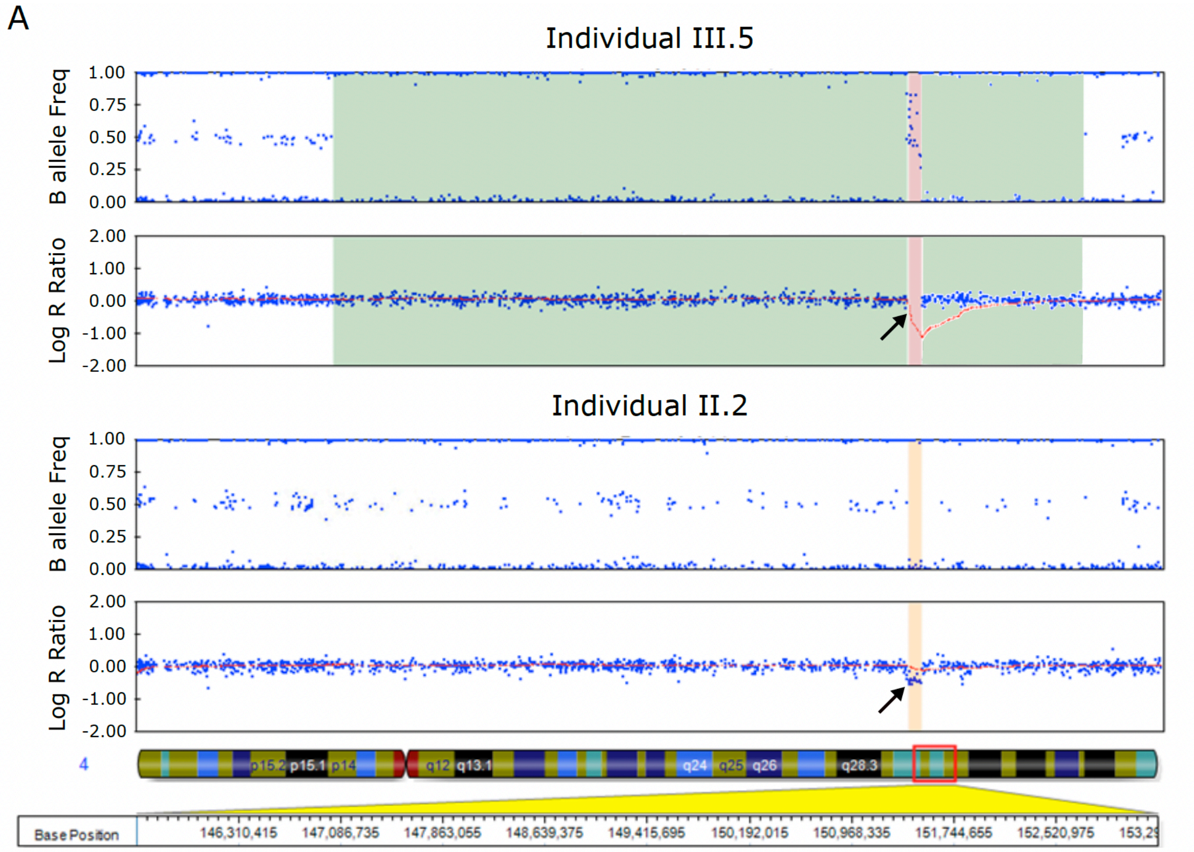

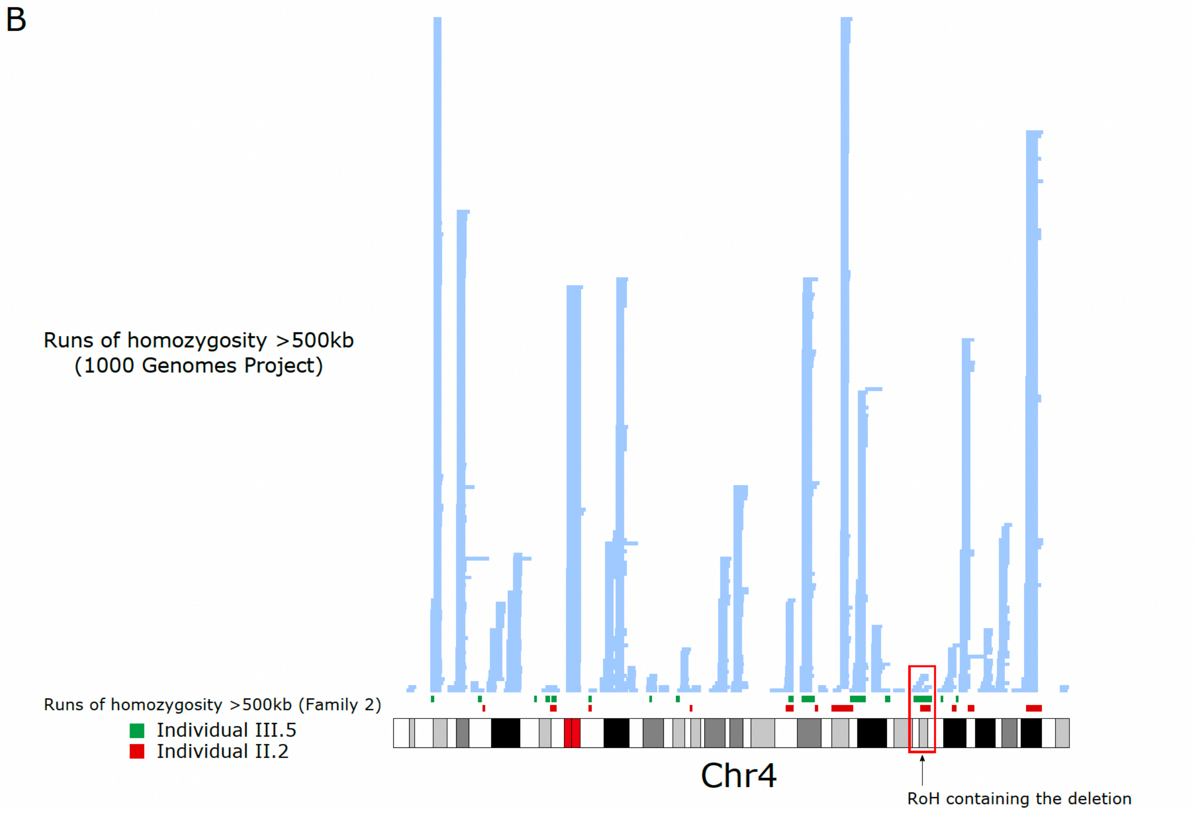

Supplementary Figure 2: Identification of 15 non-exonic conserved elements within the 113.58kb deletion of Individual III.5 (Family 2).

A: Adapted screenshot from the UCSC Genome Browser (Human Genome, hg19) indicating the position of the 15 identified conserved elements (CEs) relative to the deletion in individual III.5. Position of the CEs relative to the 100 vertebrate conservation track are indicated by red boxes. CE numbering indicated below the UCSC Genome Browser image. Red bar: region of the deletion; black bars: conserved elements. CEs 7, 8, and 10 (red) contain regulatory regions Ma, Mb and Mc, respectively, identified by Tsang *et al.*[3] **B:** Schematic representation of the deleted region upstream of *MAB21L2* in Individual III.5, and the equivalent regions in model species. *MAB21L2* is indicated in blue, and the area affected by the CNV by a horizontal red line. The locations of the identified CEs are shown using vertical lines, with those containing the elements identified by Tsang *et al.*[3] in yellow, and CE14 containing the OTX2 binding site in green.

Supplementary Figure 2

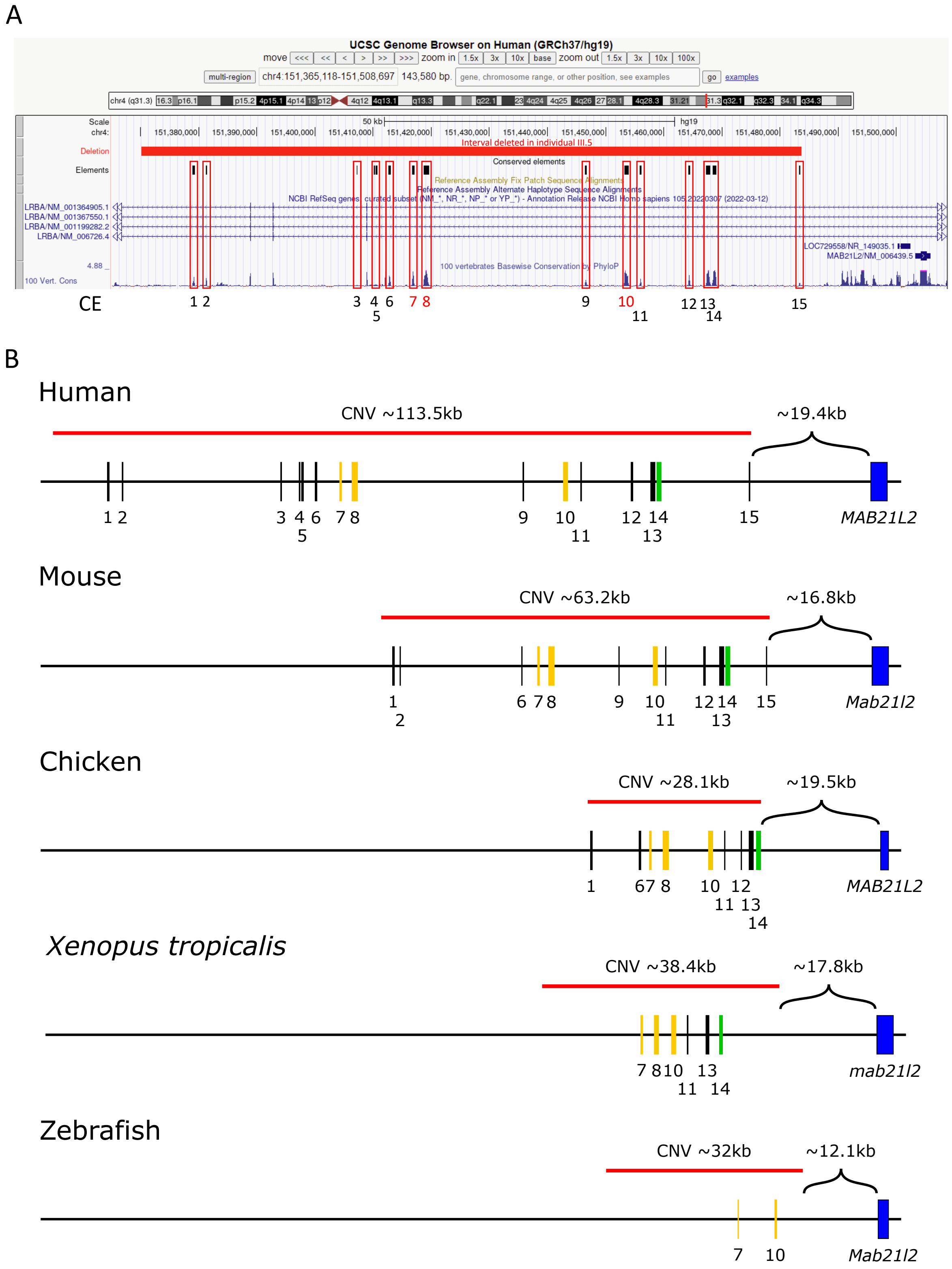

Supplementary Figure 3: Histograms of relative fold change in expression of *mab21l2* in wild-type and homozygous *mab21l2*^{mw715} fish.

The results show a decrease in *mab21l2* levels at 24-, 48- and 72hpf in homozygous eyes (Hom) which was not statistically significant (p = 0.1545, p = 0.4053, p = 0.0844, respectively) (**A**); a significant reduction in *mab21l2* levels in 48hpf (p = 0.0002;***) and 72hpf (p = 0.003;**) homozygous midbrains (**B**); and slightly diminished *mab21l2* expression at early (20- and 24hpf), but not later (48- and 72hpf) stages that was not statistically significant (p = 0.0551, p = 0.1177, p = 0.899, p = 0.7452, respectively) (**C**). The data represent the mean ± SEM (indicated with error bar) of 5 biological replicates for 24hpf eyes, 4 biological replicates for 48- and 72hpf eyes and 48hpf wild-type midbrain, and 3 biological replicates for all other experiments, with each reaction performed in triplicate. Statistical analysis was performed using a two-tailed unpaired t-test. Black bars represent wild-type, while grayscale bars represent homozygous *mab21l2*^{mw715} embryos: 20hpf (light gray); 24hpf (medium gray); 48hpf (gray); and 72hpf (dark gray). Source data are provided in the "Source Data for Supplementary Fig. 3" file.

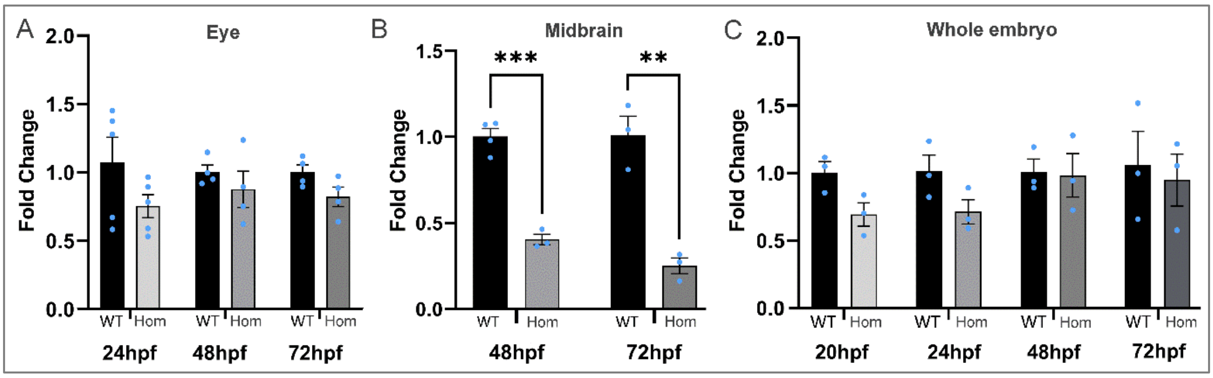

Supplementary Figure 4: ChIP-seq data from murine stem cells showing OTX2 binding of Conserved Elements 13 and 14.

A: Adapted screenshot from the UCSC Genome Browser (Mouse Genome, mm10) showing the locations of murine OTX2 ChIP-seq peaks within the Mouse genomic region equivalent to the deletion identified in Individual III.5 (Family 2) and the locations of the identified CEs. The OTX2 binding peaks for CE13 and CE14 are indicated with arrows. Note that murine *Mab21l2* is encoded on the opposite strand compared to humans and that CEs 3-5 are not conserved in the mouse. **B-C:** Adapted screenshots from the Integrative Genomics Viewer visualization tool showing mouse ChIP-seq data for CE13 (B) and CE14 (C). OTX2 binding is shown in comparison to SOX2 and IgG (control). Specific regions of binding are indicated by a red box.

Supplementary Figure 4

A

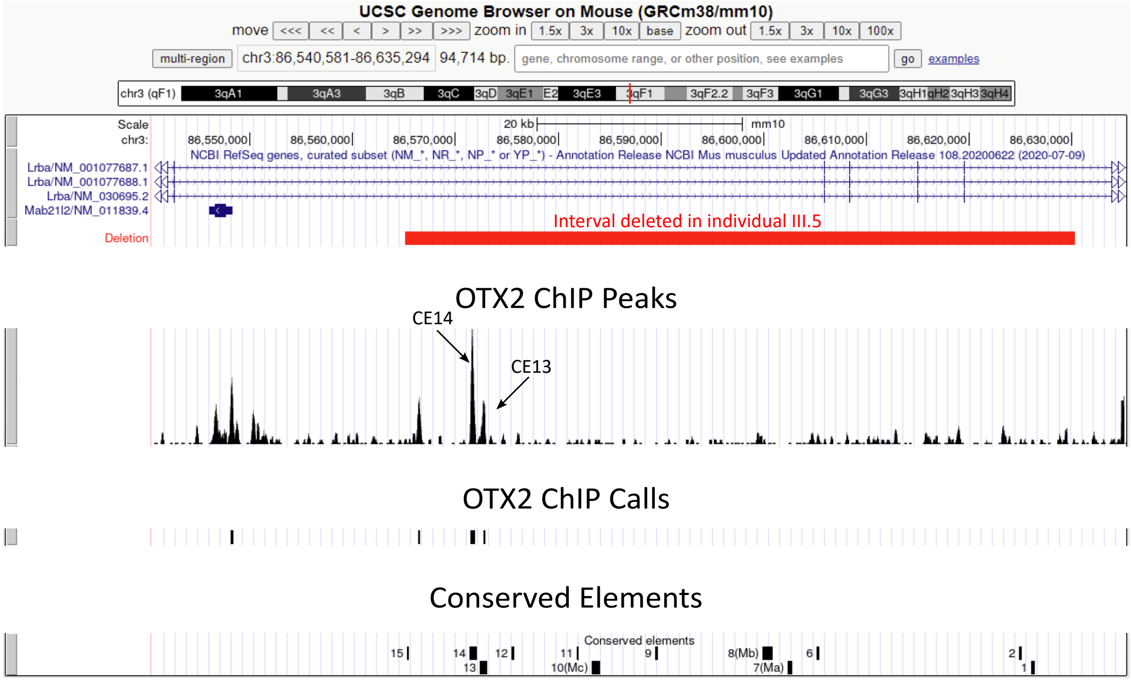

B

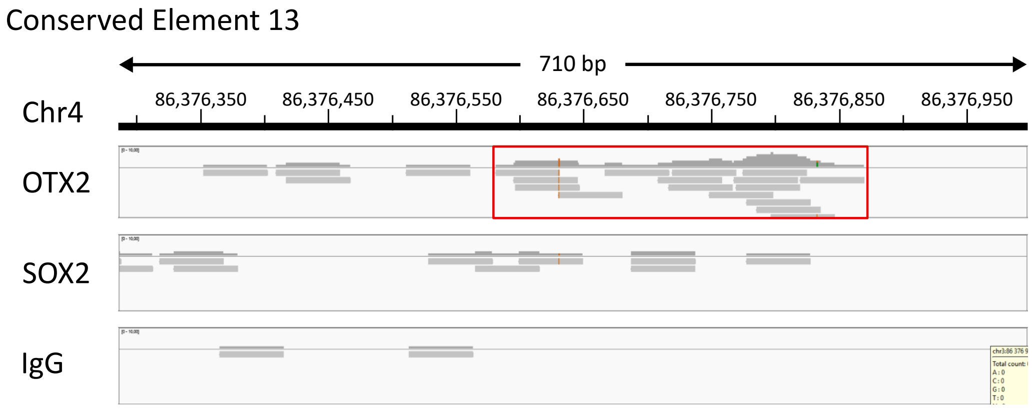

C

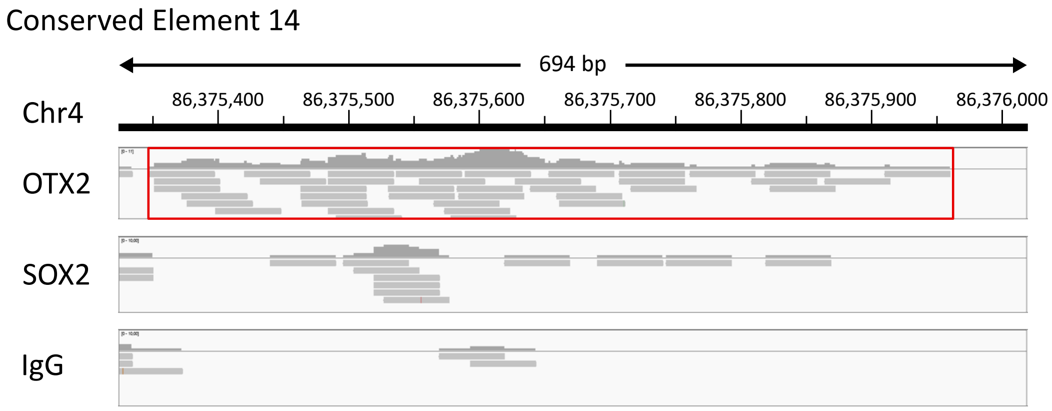

Supplementary Figure 5: Evidence of regulatory elements within the region of the deletion identified in Family 2.

A: UCSC genome browser representation of the deleted region in the human genome highlighting the deleted interval (grey), conserved elements (CEs) (purple: previously described ultra-conserved elements Ma, Mb and Mc; green: CE14, the newly identified highly conserved putative enhancer; pink: remaining 11 CEs identified in this study), and *MAB21L2* (orange). UCSC tracks indicating features typical of transcriptional enhancers (H3K4me1 and H3K27ac peaks) are shown. Sequence alignment of the predicted OTX2 binding site in human, mouse and *Xenopus tropicalis* is shown beneath, with the consensus binding sequence highlighted by a red box. **B:** UCSC genome browser session (*Xenopus* tropicalis assembly xenTro9) integrating the transcription factor (TF) binding sites, ChromHMM chromatin state predictions at NF stages 10.5 and 12.5, ATAC-seq data generated from whole embryonic *Xenopus* embryos at NF stages 10.5, 12.5, and 16 extracted from Xenbase, and conservation tracks. Location of CE14 is highlighted in grey and overlaps a conserved region with ATAC-seq peaks at NF stages 12.5 and 16 (indicating open chromatin state) and a putative otx2 binding site (black arrow).

Supplementary Figure 5

A

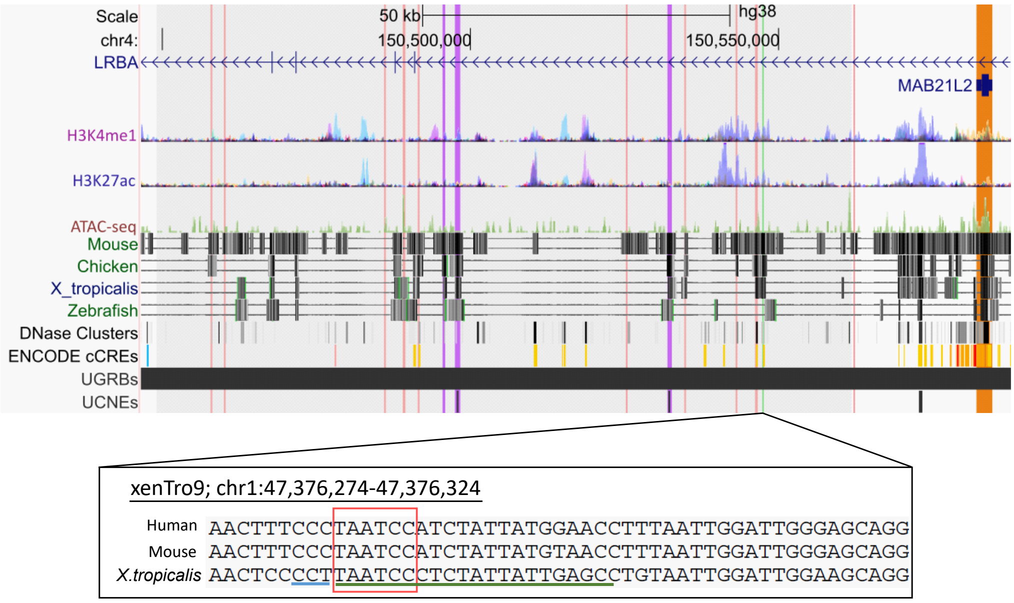

B

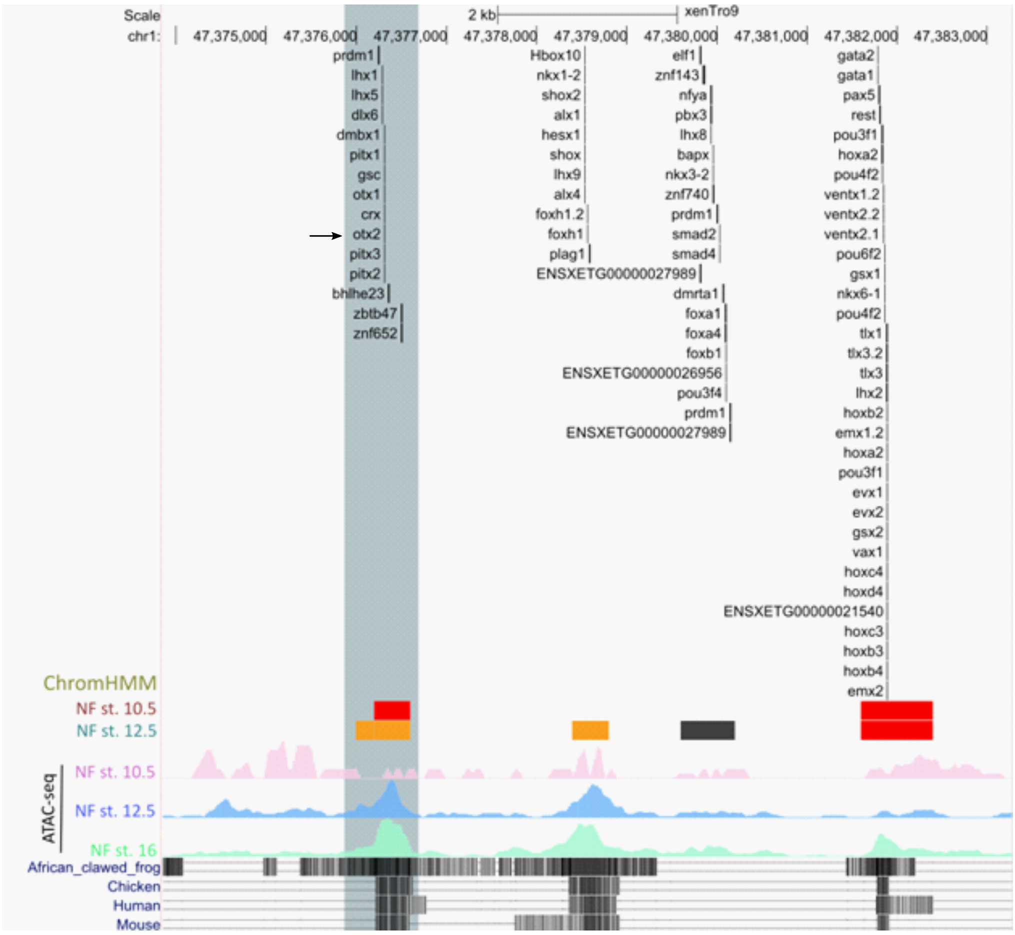

Supplementary Figure 6: CRISPR/Cas9 genome editing strategy used to generate mosaic mutant *Xenopus tropicalis* tadpoles.

A: Design of the guide RNAs used to generate a deletion of the genomic region in *Xenopus tropicalis* corresponding to the CNV detected in Family 2 (upper panel) and disruption of an Otx2 binding motif in a putative enhancer controlling *mab21l2* expression (lower panel). Panel A created with BioRender.com, released under a Creative Commons Attribution-NonCommercial-NoDerivs 4.0 International license. **B:** Confirmation of the 39kb deletion in *Xenopus tropicalis* whole embryos (NF stage 41). DNA samples extracted from each experimental group were subjected to PCR amplification of the targeted region using primers flanking the paired guide RNA target sites. Notably, the presence of the deletion in *mab21l2-5'del* animals enabled the amplification of a distinct 500bp PCR fragment. In contrast, the *mab21l2-CE14*, *mab21l2-non-CE*, and wild-type (WT) groups exhibited no amplification, corroborating the specificity and efficacy of our targeted deletion strategy. **C:** Representative analysis of the relative contribution of each indel in *mab21l2-non-CE* (upper panel) and *mab21l2-CE14* (lower panel) crispants as observed via deep amplicon sequencing. The PAM site is indicated in red and underlined.

Supplementary Figure 6

A

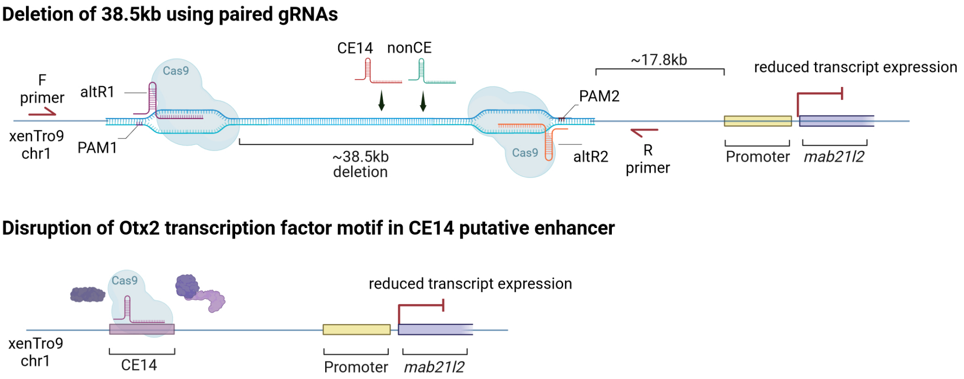

B

	Sequence	Coordinates
Forward primer	TGCTGCTGTACGGAGAATCA	chr1:47,366,776-47,366,795
Reverse primer	GCCTAGCCTATGGGTAAGAAGC	chr1:47,405,623-47,405,644

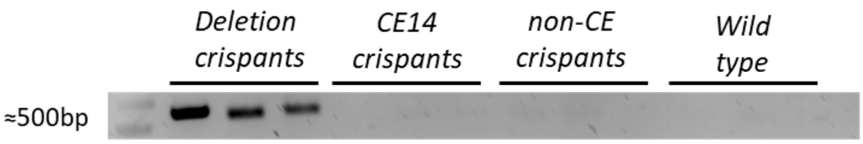

C

- **non-CE crispants editing outcome efficiency: 80%**

Indel	Contribution	Sequence	
		TCTGCTGCTGCTGCTGTACGGAGAATCAGCGCGCAAAGGATGG------NGGGGGGTCCT	
Del11	20%	TCTGCTGCTGCTGCTGTACGGAGAATCAGCGCGCAAAGGATGG------------GTCCT	48
Del9	6%	TCTGCTGCTGCTGCTGTACGGAGAATCAGCGCGCAAAGGATGG----------GGGTCCT	50
Ins1	5%	TCTGCTGCTGCTGCTGTACGGAGAATCAGCGCGCAAAGGATGGGTC-GGGGGGGGGTCCT	59
Del5	4%	TCTGCTGCTGCTGCTGTACGGAGAATCAGCGCGCAAAGGATGG------GGGGGGGTCCT	54
Del4	3%	TCTGCTGCTGCTGCTGTACGGAGAATCAGCGCGCAAAGGATGG-----GTGGGGGGTCCT	55
Del6	3%	TCTGCTGCTGCTGCTGTACGGAGAATCAGCGCGCAAAGGATGG-------GGGGGGTCCT	53
Del1	2%	TCTGCTGCTGCTGCTGTACGGAGAATCAGCGCGCAAAGGATGGGTC--GGGGGGGGTCCT	58
Del12	2%	TCTGCTGCTGCTGCTGTACGGAGAATCAGCGCGCAAAGGATGG-------------TCCT	47
Del4	2%	TCTGCTGCTGCTGCTGTACGGAGAATCAGCGCGCAAAGGATGG-----GGGGGGGGTCCT	55
Del3	2%	TCTGCTGCTGCTGCTGTACGGAGAATCAGCGCGCAAAGGATGG----GGGGGGGGGTCCT	56
Del12	2%	TCTGCTGCTGCTGCTGTACGGAGAATCAGCGCGCAAAGGGGGG-------------TCCT	47
Del7	1%	TCTGCTGCTGCTGCTGTACGGAGAATCAGCGCGCAAAGGATGG--------GGGGGTCCT	52
Del5	1%	TCTGCTGCTGCTGCTGTACGGAGAATCAGCGCGCAAAGGATGG------GTGGGGGTCCT	54
Del13	1%	TCTGCTGCTGCTGCTGTACGGAGAATCAGCGCGCAAAGG--------------GGGTCCT	46
Del3	1%	TCTGCTGCTGCTGCTGTACGGAGAATCAGCGCGCAAAGGATGG----GTGGGGGGGTCCT	56

- **CE14 crispants editing outcome efficiency: 45%**

Indel	Contribution	Sequence	
		ATTTTATATACCACCCTGCTTCCAATCCAATTACAGGCTCAATAATAG------NNNAAGGGGAG	
Del8	16%	ATTTTATATACCACCCTGCTTCCAATCCAATTACAGGCTCAATAATAG----------AGGGGAG	55
Del9	9%	ATTTTATATACCACCCTGCTTCCAATCCAATTACAGGCTCAATAATAG-----------AGGGAG	54
Ins1	3%	ATTTTATATACCACCCTGCTTCCAATCCAATTACAGGCTCAATAATAGAGGGA-NTTAAGGGGAG	64
Ins2	2%	ATTTTATATACCACCCTGCTTCCAATCCAATTACAGGCTCAATAATAGAGGGANNTTAAGGGGAG	65
Del8	2%	ATTTTATATACCACCCTGCTTCCAATCCAATTACAGGCTCAATA----------ATTAAGGGGAG	55
Del4	2%	ATTTTATATACCACCCTGCTTCCAATCCAATTACAGGCTCAATAATAG------AGGGAGGGGAG	59
Del9	1%	ATTTTATATACCACCCTGCTTCCAATCCAATTACAGGCTCAATAAT----------AAGGGGAG	54
Del4	1%	ATTTTATATACCACCCTGCTTCCAATCCAATTACAGGCTCAATAATAG------ATTAAGGGGAG	59
Ins1	1%	ATTTTATATACCACCCTGCTTCCAATCCAATTACAGGCTCAATAATAGAGGGN-ATTAAGGGGAG	64
Del10	1%	ATTTTATATACCACCCTGCTTCCAATCCAATTACAGGCTCAATAAT-----------AGGGGAG	53

Supplementary Figure 7: Comparison of retinal layering in a *CE14* crispant injected and non-injected eyes.

Histological sections stained with hematoxylin and eosin (H&E) showing preserved retinal architecture in both retinas, while a malformed lens is present on the injected side.

CE14 crispant
Non-injected side

CE14 crispant
Injected side

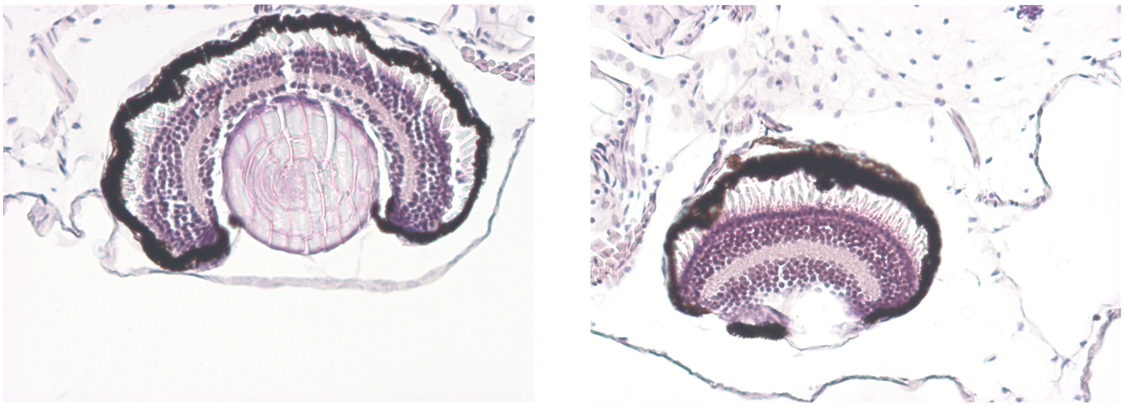

Supplementary References

1. Matías-Pérez, D. *et al.* Identification of novel pathogenic variants and novel gene-phenotype correlations in Mexican subjects with microphthalmia and/or anophthalmia by next-generation sequencing. *J Hum Genet* **63**, 1169-1180 (2018).
2. Szczot, M., Nickolls, A.R., Lam, R.M. & Chesler, A.T. The Form and Function of PIEZO2. *Annu Rev Biochem* **90**, 507-534 (2021).
3. Tsang, W.H., Shek, K.F., Lee, T.Y. & Chow, K.L. An evolutionarily conserved nested gene pair - Mab21 and Lrba/Nbea in metazoan. *Genomics* **94**, 177-87 (2009).

Supplementary Note

Supplementary Figure 6B (uncropped gel image).

The first lane shows the DNA molecular weight marker SmartLadder MW-1700-10 (Eurogentec, Belgium), with 14 bands ranging from 200bp to 10,000bp; the following 12 lanes show the result of the PCR amplification performed using primers flanking the paired gRNA target sites used to generate the 39kb deletion. The presence of a 500bp PCR fragment in *mab21l2-5'del* animals demonstrates the presence of the deletion in this group.

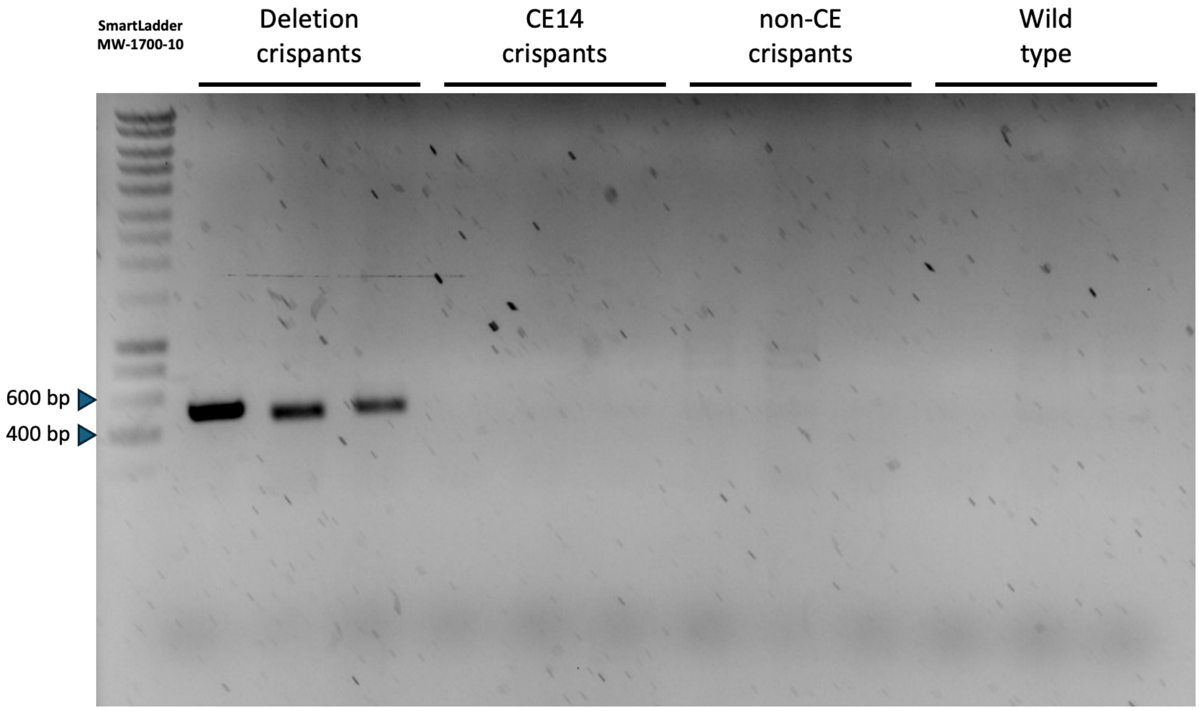

Supplement: Supplementary file 1 — Supplementary Information [file 41467_2024_53553_MOESM1_ESM.pdf]
