# Supplementary material for: Deletion upstream of MAB21L2 highlights the importance of evolutionarily conserved non-coding sequences for eye development
Source: Nat Commun. 2024 Oct 26;15:9245. doi: 10.1038/s41467-024-53553-2 (PMC11511899; doi:10.1038/s41467-024-53553-2)
Supplement: Supplementary file 2 — Description of Additional Supplementary Files [file 41467_2024_53553_MOESM2_ESM.pdf]

Description of Additional Supplementary Files

File Name: Supplementary Movie 1

Description: Three-dimensional view of Xenopus embryos bilaterally microinjected with CE14 and non-CE gRNA complexes, indicated here as CRE14 crispants and non-CRE crispants, respectively. By utilizing multiclass U-Net deep learning, we were able to reconstruct lens and retinal structures of these embryos. These structures were visualized using triple fluorescent staining for Atp1a1 (renal tubules and neural tissue), the lectins Peanut Agglutinin (PNA; cone matrix domains) and Wheat Germ Agglutinin (WGA; matrix domains surrounding rods) and imaged using mesoSPIM light-sheet microscopy. The retinal morphology of CRE14 crispants (right) showed aberrant retinal morphology when contrasted with non-CRE crispant controls (left)

File name: "Supplementary Software 1"

Description: The zip file contains a word file (“custom_scripts-annotation and filtering.docx”) that describes the workflow used to annotate and filter variants. The file also contains the code used to perform the annotations and the required source files.
