# Supplementary material for: Deletion upstream of MAB21L2 highlights the importance of evolutionarily conserved non-coding sequences for eye development
Source: Nat Commun. 2024 Oct 26;15:9245. doi: 10.1038/s41467-024-53553-2 (PMC11511899; doi:10.1038/s41467-024-53553-2)
Supplement: Supplementary file 4 — Supplementary Software 1 [file 41467_2024_53553_MOESM4_ESM.zip › Supplementary Software 1/custom_scripts-annotation and filtering.docx]

ANNOVAR ANNOTATIONS

WES vcf files have been generated according to hg19 genome build and annotated using ANNOVAR (<https://annovar.openbioinformatics.org/en/latest/user-guide/startup/>).

Format conversion

The annovar script convert2annovar.pl converts the vcf files into avinput format.

perl convert2annovar.pl -format vcf4 sample.vcf -out sample.avinput -includeinfo

Variant annotation

The annovar script table_annovar.pl annotates each variant listed in the avinput file using information retrieved from databases specified in the command below. All the databases are located within the folder “humandb”. Note: in the command below, “sample” is a placeholder for the name of the individual.

perl table_annovar.pl sample.avinput humandb/ -buildver hg19 -out sample -remove -protocol refGene,knownGene,MT_ensGene,wgRNA,cytoBand,genomicSuperDups,avsnp150,gnomAD211_exome,gnomAD211_genome,exac03,esp6500siv2_all,esp6500siv2_ea,1000g2015aug_all,1000g2015aug_eur,clinvar_20200316,intervar_20180118,dbnsfp35a,dbscsnv11 -operation g,g,g,r,r,r,f,f,f,f,f,f,f,f,f,f,f,f -arg '-exonicsplicing',,,,,,,,,,,,,,,,, -nastring . -otherinfo

CUSTOM ANNOTATIONS

**STEP1**:
**Create an extra column at the beginning of the file with the coordinates of each variant (chr:start-end).**

cat sample.hg19_multianno.txt | awk -F\t '{print $1":"$2"-"$3}' > sample.hg19_multianno1-coords.txt

paste sample.hg19_multianno1-coords.txt sample.hg19_multianno.txt > sample.hg19_multianno1.txt

**STEP2**:

**Split the variants in multiple rows if they have multiple gene names in the RefSeq column ($8). Gene names are separated by “;”. Duplicated rows are removed with sort** -u.

cat sample.hg19_multianno1.txt | awk -F\t '{split($8,names,";"); for (i in names) print names[i] "\t" $0}' | sort -u > sample.hg19_multianno2_nodupl.txt

**STEP3**: Tiger panel genes

**Annotate whether the variant affects a gene present in the Tiger Panel (a custom panel of eye candidate genes).**

The list includes genes considered as known eye genes or potential candidates based on literature searches and can be found in the file “Tiger_genes_2020.txt”.

awk -F\t '{i=$1} NR==FNR{A[i]=$0; next} {if (i in A) {print $0 "\t" "panel_refseq"} else {print $0 "\t" "NA"}}' /humandb/Tiger_genes_2020.txt sample.hg19_multianno2_nodupl.txt > sample.hg19_multianno3.txt

**STEP4**: AMC gene list

**Annotate whether the variant affects a gene present in the AMC gene list.**

The AMC list includes genes from “Structural eye disease” PanelApp v1.5 (https://panelapp.genomicsengland.co.uk/panels/509/) [labelled as green/amber/red according to the Genomics England classification] + additional genes included as potentially diagnostic by the Ragge research group [labelled as diagnostic_Ragge]. This list was generated on 30^th^ March 2020 and can be found in the file “AMC_genes_20200330.txt”.

awk -F\t '{i=$1} NR==FNR{A[i]=$2; next} {i=$1; if (i in A) {print $0 "\t" A[i]} else {print $0 "\t" "NA"}}' /humandb/AMC_genes_20200330.txt sample.hg19_multianno3.txt > sample.hg19_multianno4.txt

**STEP5**:

**Replace the spaces with underscore in the whole document.**

sed 's/ /_/g' sample.hg19_multianno4.txt > sample.hg19_multianno5.txt

**STEP6**: OMIM database

**Add gene-based annotations obtained from OMIM (**[**https://www.omim.org/**](https://www.omim.org/)**). The annotations have been retrieved from the file “genemap2.txt”, downloaded from OMIM in March 2020 (https://www.omim.org/downloads).**

The layout of the genemap2.txt file has been edited to add the following columns (14 fields) at the end of each row: Approved_gene_symbol_HGNC, Gene_symbols_OMIM, Gene_name_OMIM, Phenotypes_OMIM, Mouse_gene_symbol_&_ID_MGI, Comments_OMIM, Chromosome_NCBI, Genomic_start_GRCh38_NCBI, Genomic_end_GRCh38_NCBI, Cyto_location_OMIM, Computed_cyto_location_UCSC, MIM_Number_for_Gene/Locus_OMIM, Entrez_gene_ID_NCBI, Ensembl_gene_ID_Ensembl.

If the variant affects a gene with no annotations in OMIM_genemap2_2020.txt, 14 tab-separated “NA” are added instead.

awk -F\t '{i=$1} NR==FNR{A[i]=$0; next} {if (i in A) {print $0 "\t" A[i]} else {print $0 "\t" "NA" "\t" "NA" "\t" "NA" "\t" "NA" "\t" "NA" "\t" "NA" "\t" "NA" "\t" "NA" "\t" "NA" "\t" "NA" "\t" "NA" "\t" "NA" "\t" "NA" "\t" "NA"}}' /humandb/OMIM_genemap2_2020.txt sample.hg19_multianno5.txt > sample.hg19_multianno6.txt

**STEP7**: International Mouse Phenotyping Consortium (IMPC) database

**Add gene-based annotations obtained from IMPC (**[**https://www.mousephenotype.org/**](https://www.mousephenotype.org/)**) at the end of each row: the HGNC gene name and the Mammalian Phenotype ontology term names associated with that gene.** If the variant affects a gene with no annotations in IMPC_release9_2020.txt, 2 tab-separated “NA” are added instead.

The IMPC annotations have been retrieved from the file ALL_genotype_phenotype.csv.gz (IMPC Data Release 9), downloaded in February 2020. The edited file is called “IMPC_release9_2020.txt.

awk -F\t '{i=$1} NR==FNR{A[i]=$0; next} {if (i in A) {print $0 "\t" A[i]} else {print $0 "\t" "NA" "\t" "NA"}}' /humandb/IMPC_release9_2020.txt sample.hg19_multianno6.txt > sample.hg19_multianno7.txt

**STEP8**: ZFIN database

**Add gene-based annotations obtained from the Zebrafish database ZFIN (**[**https://zfin.org/**](https://zfin.org/)**) at the end of each row.**

The ZFIN annotations have been retrieved from the file [pheno_fish.txt](https://zfin.org/downloads/pheno_fish.txt) (“Phenotype for Zebrafish genes with Human orthology”), downloaded in March 2020. The file has been edited to keep only ZFIN Gene Symbols and Affected Structures or Processes (Superterm Name). The edited file is called “zfin_2020.txt”.

awk -F\t '{i=$1} NR==FNR{A[i]=$0; next} {if (i in A) {print $0 "\t" A[i]} else {print $0 "\t" "NA" "\t" "NA"}}' /humandb/zfin_2020.txt sample.hg19_multianno7.txt > sample.hg19_multianno8.txt

**STEP9**: ConsensusPathDB

**Add gene-based annotations obtained from the ConsensusPathDB human database (http://cpdb.molgen.mpg.de/) at the end of each row.**

The CPDB annotations have been retrieved from the file CPDB_pathways_genes-HGCN.tab downloaded in March 2020. The edited file is called “CPDB_genes-to-pathways_2020.txt”.

awk -F\t '{i=$1} NR==FNR{A[i]=$0; next} {if (i in A) {print $0 "\t" A[i]} else {print $0 "\t" "NA" "\t" "NA"}}' /humandb/CPDB_genes-to-pathways_2020.txt sample.hg19_multianno8.txt > sample.hg19_multianno9.txt

**STEP10:** gnomAD gene constraint metrics

**Add gene constraint metrics obtained from gnomAD v2.1.1.**

The metrics have been retrieved from the file gnomad.v2.1.1.lof_metrics.by_gene.vcf.bgz, downloaded in March 2020.

Ten fields from the original file have been selected for the annotations: gene, transcript, oe_mis, mis_z, pLI, oe_lof, gene_id, transcript_level, cds_length**,** num_coding_exons. The edited file is called “gnomad211-metrics_20200330.txt”.

awk -F\t '{i=$1} NR==FNR{A[i]=$0; next} {if (i in A) {print $0 "\t" A[i]} else {print $0 "\t" "NA" "\t" "NA" "\t" "NA" "\t" "NA" "\t" "NA" "\t" "NA" "\t" "NA" "\t" "NA" "\t" "NA" "\t" "NA"}}' /humandb/gnomad211-metrics_20200330.txt sample.hg19_multianno9.txt > sample.hg19_multianno10.txt

**STEP11:** Heading

**Replace the column headings initially added by** table_annovar.pl **with a line including headings for the customised annotations.**

The first column of “heading.txt” starts with “#Gene.refGene” rather than “Gene.refGene” to distinguish it from the old heading**.**

cat heading.txt sample.hg19_multianno10.txt | awk -F"\t" '($1!="Gene.refGene") {print}' > sample.hg19_multianno11.txt

VARIANT FILTERING

**FILTER 1: selection of exonic variants**

Select only the variants affecting “*exonic*” OR “*splicing*” OR “*exonic*;*splicing*” regions according to RefSeq (column $8) OR UCSC knownGene annotations (column $13).

awk -F"\t" '(/^#Gene.refGene/||$8 ~/exonic|splicing/ || $13 ~/exonic|splicing/)' sample.hg19_multianno11.txt > sample.hg19_multianno11_F1-exonic.txt

**FILTER 2: exclusion of synonymous variants**

Exclude the variants that are defined as *synonymous_SNVs* according to both RefSeq (column 11) and UCSC knownGene (column 16) and keep exonic variants that do not cause an amino acid change but might affect the splicing (defined as “exonic;splicing” in column 8 or 13 & “synonymous_SNVs” in column 11 or 16) and keep intronic variants in splicing regions.

awk -F"\t" '(/^#Gene.refGene/||$8~/splicing/ || $13~/splicing/ || ($8=="exonic" && $11!="synonymous_SNV") || ($13=="exonic" && $16!="synonymous_SNV"))' sample.hg19_multianno11_F1-exonic.txt > sample.hg19_multianno11_F2-synon-excl.txt

**FILTER 3**: **selection of rare variants**

Use minor allele frequency (MAF) reported by public databases to select rare variants.

FILTER 3a keeps variants with Alt Allele Frequency ≤ **1%** in gnomAD_exome_ALL and in gnomAD_genome_ALL and in Exac_ALL and in 1000g2015aug_all, including variants that are absent in these databases (indicated with a dot).

🡒 useful to analyse variant according to a recessive model.

awk -F"\t" '(/^#Gene.refGene/||$27 <= 0.01 && $44 <= 0.01 && $61 <= 0.01 && $71 <= 0.01)' sample.hg19_multianno11_F2-synon-excl.txt > sample.hg19_multianno11_F3-freq1%.txt

FILTER 3b keeps variants with Alt Allele Frequency ≤ **0.5%** in gnomAD_exome_ALL and in gnomAD_genome_ALL and in Exac_ALL and in 1000g2015aug_all, including variants that are absent in these databases (indicated with a dot).

🡒 useful to analyse variant according to a dominant model.

awk -F"\t" '(/^#Gene.refGene/||$27 <= 0.005 && $44 <= 0.005 && $61 <= 0.005 && $71 <= 0.005)' sample.hg19_multianno11_F2-synon-excl.txt > sample.hg19_multianno11_F3-freq0.5.txt

**FILTER 4**: **selection of homozygous variants (MAF 1%)**

Keep variants where the genotype is listed as 1/1 or 1|1 in the genotype column ($188).
Note: the character “/” needs an escape character, which is the back slash (\).

awk -F"\t" '(/^#Gene.refGene/|| $188 ~/1\/1:/ || $188 ~/1\|1:/)' sample.hg19_multianno11_F3-freq1%.txt > sample.hg19_multianno11_F4-homoz1%.txt

**FILTER 5**: **selection of heterozygous variants (MAF 0.05%)**

Keep variants that are NOT listed as 1/1 or 1|1 in the genotype column ($188).

awk -F"\t" '(/^#Gene.refGene/|| $188 !~/1\/1:/ && $188 !~/1\|1:/)' sample.hg19_multianno11_F3-freq0.5.txt > sample.hg19_multianno11_F5-het0.5.txt

**FILTER 6**: **selection of ultra-rare variants**

From the list of heterozygous variants with MAF≤ 0.5%, extract those with Alt allele frequency ≤0.1% in gnomAD_exome_ALL and in gnomAD_genome_ALL and in Exac_ALL and in 1000g2015aug_all.

awk -F"\t" '(/^#Gene.refGene/||$27 <= 0.001 && $44 <= 0.001 && $61 <= 0.001 && $71 <= 0.001)' sample.hg19_multianno11_F5-het0.5.txt > sample.hg19_multianno11_F6-freq0.1.txt

**FILTER 7**: **selection of likely damaging ultra-rare variants**

**Step 1** keeps the lines that contain the word *splicing* in RefSeq ($8) or UCSC knownGene ($13) OR *frameshift* or *stop* in RefSeq ($11) or UCSC knownGene ($16) annotations.

awk -F"\t" '(/^#Gene.refGene/||$8 ~/splicing/ || $13 ~/splicing/ || $11 ~/frameshift|stop/ || $16 ~/frameshift|stop/) {print}' sample.hg19_multianno11_F6-freq0.1.txt > sample.hg19_multianno11_F7_step1.txt

**Step 2** keeps the lines that contain the word *nonsynonymous* in RefSeq ($11) or UCSC knownGene ($16)**.**

awk -F"\t" '($11 ~/nonsynonymous/ || $16 ~/nonsynonymous/){print}' sample.hg19_multianno11_F6-freq0.1.txt > sample.hg19_multianno11_F7_step2.txt

**Step 3** keeps the *nonsynonymous* variants for which the prediction of MetaSVM_pred ($135) OR MetaLR_pred ($138) is damaging OR CADD_phred≥15 ($148).

awk -F"\t" '($135!="T"|| $138!="T" || $148>=15){print}' sample.hg19_multianno11_F7_step2.txt > sample.hg19_multianno11_F7_step3.txt

**Step 4** collates the files generated by the previous steps in one file and removes duplicates rows:

cat sample.hg19_multianno11_F7_step1.txt sample.hg19_multianno11_F7_step3.txt > sample.hg19_multianno11_F7_step4.txt

sort -u sample.hg19_multianno11_F7_step4.txt > sample.hg19_multianno11_F7_damaging.txt
