# Supplementary material for: Deletion upstream of MAB21L2 highlights the importance of evolutionarily conserved non-coding sequences for eye development
Source: Nat Commun. 2024 Oct 26;15:9245. doi: 10.1038/s41467-024-53553-2 (PMC11511899; doi:10.1038/s41467-024-53553-2)
Supplement: Supplementary file 5 — Reporting Summary [file 41467_2024_53553_MOESM5_ESM.pdf]

Corresponding author(s): Professor Nicola Ragge
Last updated by author(s): Oct 10, 2024

Reporting Summary

Nature Portfolio wishes to improve the reproducibility of the work that we publish. This form provides structure for consistency and transparency in reporting. For further information on Nature Portfolio policies, see our Editorial Policies and the Editorial Policy Checklist.

Statistics

For all statistical analyses, confirm that the following items are present in the figure legend, table legend, main text, or Methods section.

n/a	Confirmed	
☐	☒	The exact sample size (*n*) for each experimental group/condition, given as a discrete number and unit of measurement
☐	☒	A statement on whether measurements were taken from distinct samples or whether the same sample was measured repeatedly
☐	☒	The statistical test(s) used AND whether they are one- or two-sided<br>*Only common tests should be described solely by name; describe more complex techniques in the Methods section.*
☒	☐	A description of all covariates tested
☒	☐	A description of any assumptions or corrections, such as tests of normality and adjustment for multiple comparisons
☐	☒	A full description of the statistical parameters including central tendency (e.g. means) or other basic estimates (e.g. regression coefficient) AND variation (e.g. standard deviation) or associated estimates of uncertainty (e.g. confidence intervals)
☐	☒	For null hypothesis testing, the test statistic (e.g. *F*, *t*, *r*) with confidence intervals, effect sizes, degrees of freedom and *P* value noted<br>*Give P values as exact values whenever suitable.*
☒	☐	For Bayesian analysis, information on the choice of priors and Markov chain Monte Carlo settings
☒	☐	For hierarchical and complex designs, identification of the appropriate level for tests and full reporting of outcomes
☐	☒	Estimates of effect sizes (e.g. Cohen's *d*, Pearson's *r*), indicating how they were calculated

Our web collection on statistics for biologists contains articles on many of the points above.

Software and code

Policy information about availability of computer code

Data collection

Microsoft Excel 2016 for study cohort records.

Data analysis

CNV calling: PennCNV and QuantiSNP

a) PennCNV v1.0.4
PennCNV is a free software tool for Copy Number Variation (CNV) detection from SNP genotyping arrays. It can handle signal intensity data from Illumina and Affymetrix arrays.

b) QuantiSNP v2
QuantiSNP is a free tool to identify putative copy number alterations from Illumina Infinium I/II SNP genotyping data.

Detection of ROH from SNP data: PLINK v1.9
PLINK is a free toolset designed to perform a wide range of large-scale analyses of genomic data in a computationally efficient manner, including the analysis of runs of homozygosity (ROH) from SNP array data.
ROH analyses were performed using the --homozyg function. As PLINK recommends performing this analysis on sets of SNPs that have been pruned for strong local LD, the SNP data was first pruned using the function “--indep-pairwise 50 5 0.2”, then the ROHs were detected using the functions “--homozyg --homozyg-snp 50 --homozyg-kb 1000”.

Detection of ROH from WES data: custom script
A custom Python script was written to summarise VCF files generated from WES. Example population data was obtained from the 1000

Genomes Project and assessed using the same methodology. All the codes and example data are included at the indicated github repository (https://github.com/sclokie/roh).

Analysis of Family 1 sequencing data
In-house custom pipeline was used to retrieve both CNVs and SNVs from a 119 gene panel. All variants are classified manually according to the ACMG criteria.

Analysis of Family 2 sequencing data
VCF files were annotated using the free software tool ANNOVAR (version 2019). An in-house custom pipeline was then used to add custom annotations (e.g. genes present in diagnostic eye panels) and filter the variants according to minor allele frequency and functional effects, in order to prioritise rare likely pathogenic variants. The codes and read-me file are provided in a zipped folder.

GraphPad Prism 9
GraphPad is a versatile tool purpose-built for statistical analysis of both quantitative and categorical data.

Qbase+
qbasePLUS is the professional successor of the Ghent University qBase software for real-time PCR data analysis. qbasePLUS is based on the proven geNorm and qBase technology, enhanced with proprietary algorithms and innovative features. The software provides the required power and functionality for advanced users (large datasets, inter-run calibration, etc), while keeping an intuitive approach that is also suitable for non-experts (wizard guided statistical analysis, automatic expert interpretation of genormPLUS results, etc.)
In this study, qbase+ was used for qPCR data analysis (Xenopus data).

Processing of mesoSPIM recordings: U-Net Fiji plug-in
U-Net Fiji plugin can be found at following hyperlinks:
https://lmb.informatik.uni-freiburg.de/resources/opensource/unet/
https://github.com/lmb-freiburg/Unet-Segmentation

U-Net model
Pre-trained weight and modeldef files for U-Net model can be found at https://lienkamplab.org/deep-learning-models/.

Imaris
Imaris (Bitplane) is a leading software platform designed for visualizing, analyzing, and understanding microscopy data, particularly in the fields of life sciences and biomedical research. It provides advanced features for 3D data exploration, offering tools for segmentation, quantification, and presentation of complex biological information.

gRNA design: CRISPRscan (https://www.crisprscan.org)
CRISPRscan is a scoring algorithm from the Giraldez Lab (Yale University) that helps select the best gRNAs for a given gene, predicting both cutting efficiency and possible target rate.

For manuscripts utilizing custom algorithms or software that are central to the research but not yet described in published literature, software must be made available to editors and reviewers. We strongly encourage code deposition in a community repository (e.g. GitHub). See the Nature Portfolio guidelines for submitting code & software for further information.

Data

Policy information about availability of data

All manuscripts must include a data availability statement. This statement should provide the following information, where applicable:

- Accession codes, unique identifiers, or web links for publicly available datasets
- A description of any restrictions on data availability
- For clinical datasets or third party data, please ensure that the statement adheres to our policy

Participant raw sequencing and genotyping data supporting this study are not publicly available due to their containing information, which could compromise research participant privacy and consent. Informed consent in this work does not cover the deposition of full sequencing data from the participants to a repository. Access to specific subsets of processed data generated in this study can be obtained by request from Nicola Ragge (nragge@brookes.ac.uk) with a period for response to the request of one calendar month. Data can only be shared for research purposes with permission of the patient and/or legal guardian(s) and via data sharing agreements. The processed data cannot be shared with third parties; if the data is to be used for scientific presentations and/or publications, the applicant should contact Nicola Ragge for agreement, and with agreed acknowledgement.

Sanger sequencing data confirming the two variants described in this study have been deposited to SRA (https://www.ncbi.nlm.nih.gov/sra/PRJNA1009210).

The VCF files containing sequencing data generated by the 1000 Genomes Project and used in our ROH analyses are listed at https://github.com/sclokie/roh.

The data supporting the zebrafish and Xenopus tropicalis findings are available within the paper and supplementary files. Raw data underlying the graphs included in figures 2b, 2c, 3c, 3e, 3f, 4a and 4b have been provided as a Source Data file. Raw data underlying the graphs included in Supplementary Figure 3 are provided in a separate source data file (Source Data for Supplementary Figure 3). Additional data underlying Figures 2, 3 and 4 as well as Supplementary Figures 2-6 are available upon request.

The ChIP-seq data generated in this study have been deposited to the GEO database (GEO accession number: GSE241711, https://www.ncbi.xyz/geo/query/acc.cgi?acc=GSE241711; NCBI BioProject: PRJNA1009210, https://www.ncbi.nlm.nih.gov/sra/PRJNA1009210).

Pre-trained weight and model files for the U-Net model can be found at https://lienkamplab.org/deep-learning-models.

Research involving human participants, their data, or biological material

Policy information about studies with human participants or human data. See also policy information about sex, gender (identity/presentation), and sexual orientation and race, ethnicity and racism.

Reporting on sex and gender	MAB21L2 variants are autosomally inherited and therefore findings in this manuscript apply to both male and female sexes. In both the UK and French study cohorts from which the families reported in this manuscript were drawn there is an equal representation of male and female participants. As the overall number of families described in this manuscript is small (n=2) we have not performed any sex or gender specific analyses.
Reporting on race, ethnicity, or other socially relevant groupings	We have not used any race, ethnicity or other socially relevant groupings in this manuscript. Both UK and French cohorts are comprised of individuals from various ethnicities present in the UK and France. Both cohorts are slightly enriched for individuals from communities that have higher levels of consanguinity.
Population characteristics	Our study utilizes cohorts of clinically recruited families with developmental eye anomalies. These were analysed on a case by case basis and therefore population characteristics of the cohorts are not relevant to the study.
Recruitment	For the UK cohort individuals with developmental eye conditions were recruited when presenting in genetics/ opthalmology clinics. A small number of individuals were recruited through a family support organisation (MACS, https://macs.org.uk). For the French cohort, individuals with developmental eye conditions were recruited when presenting in genetics/ ophthalmology clinics.
Ethics oversight	UK participants were recruited as part of a national 'Genetics of Eye and Brain Anomalies' study (REC 04/Q0104/129); French cases gave informed consent according to French Law. Informed consent was obtained according to the tenets of the Declaration of Helsinki.

Note that full information on the approval of the study protocol must also be provided in the manuscript.

Field-specific reporting

Please select the one below that is the best fit for your research. If you are not sure, read the appropriate sections before making your selection.

☒ Life sciences ☐ Behavioural & social sciences ☐ Ecological, evolutionary & environmental sciences

For a reference copy of the document with all sections, see nature.com/documents/nr-reporting-summary-flat.pdf

Life sciences study design

All studies must disclose on these points even when the disclosure is negative.

Sample size	Human: Individual sequencing data were analysed on a case-by-case basis and all significant variants detected in MAB21L2 are listed in this manuscript. The manuscript does not draw any conclusions on the frequency of MAB21L2 variants and therefore sample size of the cohort, albeit mentioned for reference, is not relevant in this paper.<br>Xenopus: Sample sizes were chosen based on common practices in the field and previous literature on similar experiments. Each experiment included a minimum of five to six samples per group to ensure adequate representation and ensuring variability within the data. In cases where the initial sample sizes were not sufficient to draw definitive conclusions, we grouped data from independent microinjections to increase the overall sample size and performed adequate statistical analysis. While no formal statistical sample size calculation was performed, these sample sizes are considered sufficient for detecting meaningful biological differences and ensuring the robustness of the findings.<br>Zebrafish: Sample sizes were chosen based on common practices in the field.
Data exclusions	Human: Variants with low reads/poor QC metrics were excluded.<br>Xenopus: No data were excluded.<br>Zebrafish: No data were excluded for lens and eye measurements; qRT-PCR experiments with multiple peaks in a melt curve, indicative of non-specific amplification or primer-dimer formation, were excluded from further analysis, as noted in the text.
Replication	Human: NGS sequencing was performed at suitable read depth to verify legitimacy of variants identified. Variants identified in families 1 and 2 were validated using Sanger Sequencing.<br>Xenopus: To ensure the reproducibility of our experimental findings, we conducted each experiment independently at least three times, using different batches of embryos. Consistent results were obtained across all replicates, confirming the robustness and reproducibility of our data. All experimental findings were successfully replicated without any inconsistencies, demonstrating the reliability of our experimental procedures.<br>Zebrafish: Experiments were replicated as indicated in the manuscript.<br>ChIP-seq: No replication.
Randomization	Human: No randomization was used as this study is not designed as a clinical trial.<br>Xenopus: Randomization of the Xenopus experiments is not applicable.<br>Zebrafish: Randomization of the Zebrafish experiments is not applicable.<br>ChIP-seq: No randomization.
Blinding	Human: No blinding was used as this study is not designed as a clinical trial.

Blinding

Xenopus: Morphological phenotyping of the developmental malformations was scored blindly by multiple researchers.
Zebrafish: Morphological phenotyping of the developmental phenotype was scored blindly by two researchers.
ChIP-seq: No blinding.

Reporting for specific materials, systems and methods

We require information from authors about some types of materials, experimental systems and methods used in many studies. Here, indicate whether each material, system or method listed is relevant to your study. If you are not sure if a list item applies to your research, read the appropriate section before selecting a response.

Materials & experimental systems

n/a	Involved in the study	
☐	☒	Antibodies
☐	☒	Eukaryotic cell lines
☒	☐	Palaeontology and archaeology
☐	☒	Animals and other organisms
☐	☒	Clinical data
☒	☐	Dual use research of concern
☒	☐	Plants

Methods

n/a	Involved in the study	
☐	☒	ChIP-seq
☒	☐	Flow cytometry
☒	☐	MRI-based neuroimaging

Antibodies

Antibodies used

ChIP-Seq was performed according to the Upstate (Millipore) protocol, using:
- anti-OTX2 antibody (abcam, ab21990-100, 10µg)
- anti-SOX2 (Santa Cruz Biotechnology, sc-17320, 10µg)
- mouse IgG (Millipore, PP54,10µg)

Xenopus immunostaining
- mouse anti-Atp1a1 (1:200, DSHB, A5, AB_2166869)
- Wheat Germ Agglutinin, Alexa Fluor™ 594 [WGA] [1:200 ThermoFisher, W11262]
- Lectin PNA from Arachis hypogaea [peanut], Alexa Fluor™ 488 [1:200, ThermoFisher, L21409]).

Validation

All antibodies used in Xenopus have been validated in previous studies. Antibodies for ChIP-seq experiments were commercially obtained, with no further validation performed.

Eukaryotic cell lines

Policy information about cell lines and Sex and Gender in Research

Cell line source(s)

CCE-Rx cells (Tabata et al. 2004, PMC400481) were murine stem cells genetically modified to overexpress the mouse gene Rx/rax.

Briefly, a full-length cDNA fragment for mouse Rx/rax was cloned into CAG-KS, which contained the CAG promoter followed by multicloning sites derived from Blue Script.

Stable clones of feeder-free ES cells (CCE) expressing Rx/rax (CCE-RX) were established by transfection of CCE (1 × 10^7 cells) with CAG-Rx/rax in 400 µl of opti MEM (Gibco) by electroporation (0.3 kV, 250 µF using Bio-Rad Gene Pulser Xcell). Clones were selected by use of G418 (500 µg/ml) and/or hygromycin (500 µg/ml). Selected clones were screened by PCR or immunoblot analysis of the gene product of the introduced gene.

Authentication

CCE-Rx cells were gifted by Prof. S. Watanabe (Tabata et al. 2004, PMC400481).

Mycoplasma contamination

No

Commonly misidentified lines
(See ICLAC register)

No

Animals and other research organisms

Policy information about studies involving animals; ARRIVE guidelines recommended for reporting animal research, and Sex and Gender in Research

Laboratory animals

Danio rerio
Xenopus tropicalis

Wild animals	The study did not involve wild animals.
Reporting on sex	Zebrafish: the sex of the animal is not known at embryonic and larval stages.<br>Xenopus: the sex of the animal is not known at embryonic and tadpoles stages.
Field-collected samples	No field collected samples were used.
Ethics oversight	Zebrafish: All experiments were conducted in accordance with MCW Institutional Animal Care and Use Committee approved protocol (protocol number: AUA00000352_AA_18).<br>Xenopus: all experiments on Xenopus were overseen and approved by the Ethical Committee of the Faculty of Sciences at Ghent University (EC2017-104). However, ethical approval is not required for the prefeeding embryonic stages used in this manuscript.

Note that full information on the approval of the study protocol must also be provided in the manuscript.

Clinical data

Policy information about clinical studies

All manuscripts should comply with the ICMJE guidelines for publication of clinical research and a completed CONSORT checklist must be included with all submissions.

Clinical trial registration	UK participants were recruited as part of the 'Genetics of Eye and Brain Anomalies study'. This is a descriptive study and not a clinical trial and as such a clinical trial registration number is not available. The study was approved by the UK NHS regional ethics committee Cambridge-East (04/q0104/129). French patients were recruited for diagnostic purposes and not as a clinical trial.
Study protocol	The study protocol for the 'Genetics of Eye and Brain Anomalies Study' (Cambridge East Research Ethics Committee REC 04/Q0104/129) can be made available upon request.
Data collection	Neither study described in this manuscript is a clinical trial and as such this section is not applicable.
Outcomes	Neither study described in this manuscript is a clinical trial and as such this section is not applicable.

Plants

Seed stocks	No plants were used in this study.
Novel plant genotypes	No plants were used in this study.
Authentication	No plants were used in this study.

ChIP-seq

Data deposition

☒ Confirm that both raw and final processed data have been deposited in a public database such as GEO.

☒ Confirm that you have deposited or provided access to graph files (e.g. BED files) for the called peaks.

Data access links<br>*May remain private before publication.*	All data have been deposited to the GEO database (GEO accession number GSE241711):<br>https://www.ncbi.nlm.nih.gov/geo/query/acc.cgi?acc=GSE241711<br><br>The data are also accessible via the SRA BioProject PRJNA1009210:<br>https://www.ncbi.nlm.nih.gov/sra/PRJNA1009210
Files in database submission	GDZ-09-IgG.fastq.gz<br>GDZ-10-OTX2.fastq.gz<br>GDZ-13-SOX2.fastq.gz<br><br>GDZ-09-IgG.mm10.hisat2.sorted.brm.bam.default-homer-tags.ucsc.bigWig<br>GDZ-10-OTX2.mm10.hisat2.sorted.brm.bam.default-homer-tags.ucsc.bigWig<br>GDZ-13-SOX2.mm10.hisat2.sorted.brm.bam.default-homer-tags.ucsc.bigWig<br><br>GDZ-09-IgG.mm10.hisat2.sorted.brm.bam.default.s180.minDist400FDR00001.v-.bed<br>GDZ-10-OTX2.mm10.hisat2.sorted.brm.bam.default.s180.minDist400FDR00001.v-.bed<br>GDZ-13-SOX2.mm10.hisat2.sorted.brm.bam.default.s180.minDist400FDR00001.v-.bed
Genome browser session<br>(e.g. UCSC)	https://genome-euro.ucsc.edu/s/Fabiola/ChIP%2Dmm10%2D2011

Methodology

Replicates	Experiments were performed in duplicate.
Sequencing depth	An average of 10 million reads were generated per sample.
Antibodies	anti-OTX2 antibody (abcam, ab21990-100)<br>anti-SOX2 (Santa Cruz Biotechnology, sc-17320)<br>mouse IgG (Millipore, PP54)
Peak calling parameters	Sample data converted to a tag directory using homer, with default settings.<br><br>Peaks called using homer (2022 version), using -region setting with a size of 180 bases and a -minDist of 400 between peaks and a -fdr .00001.<br><br>Peaks were called using IgG as input, to improve peak calling specificity (homer)
Data quality	An average of ~20,000 peaks were called with an FDR better than 5%.
Software	The homer package was used to process the ChIP-Seq data:<br>http://homer.ucsd.edu/homer/ngs/index.html
